# Delayed Cerebral Abnormalities in Acute Hyperammonemic Encephalopathy

**DOI:** 10.7759/cureus.10306

**Published:** 2020-09-08

**Authors:** Hiroshi Ito, Yasuhiro Ogawa, Nobutake Shimojo, Satoru Kawano

**Affiliations:** 1 Division of Hospital Medicine, University of Tsukuba Hospital, Tsukuba, JPN

**Keywords:** acute hyperammonemic encephalopathy, cirrhosis

## Abstract

Acute hyperammonemic encephalopathy (AHE) is a rare but life-threatening condition. We present a case of an 81-year-old woman with cirrhotic AHE who presented with prolonged disorientation. Her magnetic resonance (MR) images were normal on the third hospital day, which showed bilateral abnormalities in the insular and cingulate cortices on day 13. The imaging abnormalities were slightly improved but remained on day 24. The imaging abnormalities seemed correlated with her persistent disorientation. AHE can present as delayed cerebral abnormalities, and follow-up imaging tests are useful in detecting such conditions. Further reports are needed to investigate the correlation between imaging abnormalities and clinical outcomes in patients with AHE.

## Introduction

Patients with acute hyperammonemic encephalopathy (AHE) present with impaired consciousness, seizures, and death due to the toxic effect of ammonia on the brain [1]. AHE can be caused by hepatic disorders, urea cycle disorders, and drugs, including antiepileptics [2].

The typical radiologic findings of AHE have been known as four different types: diffuse cerebral edema, extensive infarct-like abnormalities, ischemic lesions, and symmetric cortical involvement [3]. However, little is known about the time course of changes in imaging findings of AHE.

Here we describe a patient with cirrhotic AHE who presented with persistent disorientation. Her magnetic resonance (MR) images of the brain were normal on admission, which later showed bilateral cortical abnormalities.

## Case presentation

An 81-year-old Japanese woman with hepatitis B virus-related cirrhosis admitted to our hospital because of impaired consciousness. Her heart rate was 103 beats per minute, blood pressure 154/72 mmHg, temperature 98℉, and respiratory rate 16 per minute. Neurological examination did not reveal neck rigidity and abnormal deep tendon reflexes. She was suspected of having hepatic encephalopathy because of asterixis and received branched-chain amino acids and lactulose. The plasma ammonia level was 322 μg/dL as seen in Table 1, and cerebrospinal fluid polymerase chain reaction for herpes simplex virus was negative.

**Table 1:** Laboratory data of the blood samples Serum ammonia reached its peak on the third hospital day, and decreased gradually.

	Day 1	Day 2	Day 3	Day 4	Day 5	Day 10	Day 15	Day 23	Day 41	Day 51
White blood cell (/μL)	11,100	17,000	20,500	17,300	16,800	7,700	8,600	7,800	8,000	6,600
Hemoglobin (g/dL)	7.2	6.6	9.7	8.6	8	8.3	8.8	8.7	7.8	8.4
Platelet (×10⁴ /μL)	21.6	24.4	23.6	18.8	11.7	9.9	12.9	9.1	10.4	10.7
Asparete aminotransferase (U/L)	22	30	29	73	95	132	70	38	28	37
Alanine aminotransferase (U/L)	12	12	13	26	81	133	102	41	17	15
Lactate dehydrogenase (U/L)	302	383	494	622	456	468	553	632	411	446
Alkaline phosphatase (U/L)	189		185	152	174	741	739	595		
γ-glutamyl transferase (U/L)	40	37	35	41	60	378	321	226	122	
Sodium (mEq/L)	140	142	148	150	161	146	153	155	147	150
Chlorine (mEq/L)	98	101	106	114	129	115	118	121	110	112
Pottasium (mEq/L)	2.8	3.1	3	4	3.6	3.8	3.1	4	4.2	4.9
Urea nitrogen (mg/dL)	80.1	93.3	131.1	153.1	125.6	32.3	42.2	31.5	40	30.5
Creatinine (mg/dL)	0.95	1.22	1.92	1.81	1.15	0.94	0.81	0.81	0.73	0.66
Ammonia (μg/dL)		322	417	214	115	54	42		57	
C-reactive protein (mg/dL)	2.2	2.24	3.32	4.63	3.94	1.8	2.91	0.97	3.21	1.84

**Figure 1:**
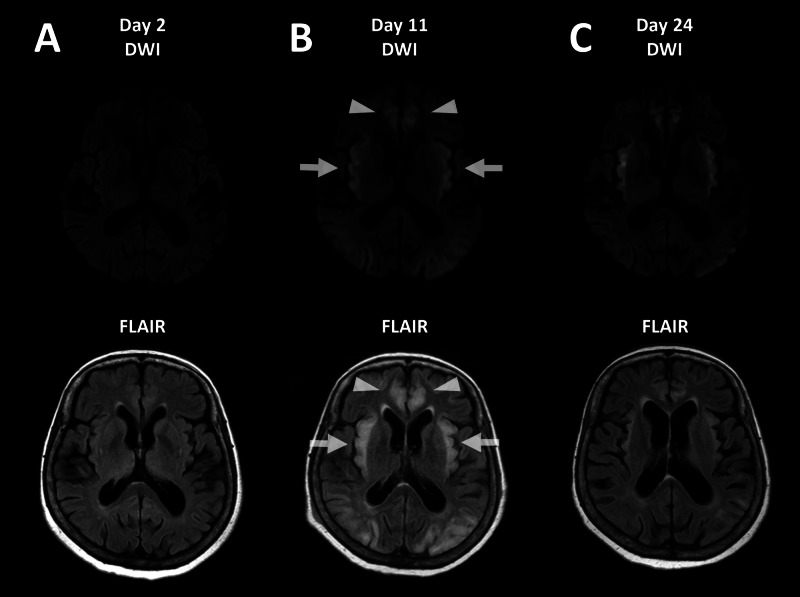
MR images of the brain (A) Initial MR images of the brain showing no remarkable changes. (B) MR images on day 11 showing symmetric abnormal signal intensity in the insular (arrows) and cingulate cortices (arrowheads) on diffusion-weighted imaging (DWI) and fluid-attenuated inversion recovery (FLAIR) imaging sequences. (C) MR images on day 24 showing improvement of the abnormal signal intensity.

Initial MR images of the brain showed no remarkable changes (Figure 1A). Electroencephalogram revealed triphasic waves.

She developed status epilepticus and was intubated on the third hospital day. MR images on day 11 showed symmetric abnormal signal intensity in the insular and cingulate cortices bilaterally, which suggested the toxic effect of accumulated ammonia (Figure 1B).

Her consciousness improved slightly after extubation on day 13, when the plasma ammonia level was 32 μg/dL. The abnormal signal intensity on the brain MR images partially improved on day 24, but her disorientation remained (Figure 1C). She was transferred to a long-stay hospital to continue rehabilitation on day 52.

## Discussion

We found out two important clinical issues. AHE can present as delayed cerebral abnormalities. Follow-up MR imaging is useful for the diagnosis of this condition.

First, AHE can present as late-onset abnormalities of the brain. Previous reports have described various radiographic findings of AHE (Table 2).

**Table 2:** Previous reports on hyperammonemic encephalopathy MR- magnetic resonance, HCV- hepatitis C virus, OTC- ornithine transcarbamylase, HBV- hepatitis B virus

Author	Case	Age	Gender	Etiology	Plasma ammonia	MR imaging finding
Gomceli YB, et al. (2007) [4]	1	20	female	drug-induced liver injury (valproic acid)	133 mg/dL	right mesial temporal sclerosis
	2	23	female	drug-induced liver injury (valproic acid)	132 mg/dL	cerebellar agenesis
	3	28	male	drug-induced liver injury (valproic acid)	130 mg/dL	postoperative changes (related to the brain surgery)
	4	56	male	drug-induced liver injury (valproic acid)	115 mg/dL	lacunar infarcts
	5	55	female	drug-induced liver injury (valproic acid)	124 mg/dL	normal
	6	64	female	drug-induced liver injury (valproic acid)	142 mg/dL	normal
	7	39	male	drug-induced liver injury (valproic acid)	122 mg/dL	normal
Velioğlu SK, et al. (2007) [5]	8	19	male	drug-induced liver injury (valproic acid)	119 μg/dL	not available
U-King-Im JM, et al. (2011) [6]	9	24	female	post-heart-lung transplant	286 μg/dL	bilateral changes in the insular and cingulate cortices
	10	48	male	drug-induced liver injury (acetaminophen)	173 μg/dL	bilateral canges in the insular and cingulate cortices, and thalami
	11	55	male	HCV-related cirrhosis	139 μg/dL	bilateral changes in the insular and cingulate cortices
	12	42	female	post-liver transplant graft rejection	94 μg/dL	bilateral changes in the insular and cingulate cortices
Tarafdar S, et al. (2011) [7]	13	36	male	drug-induced liver injury (valproic acid)	483 μg/dL	not available
Treusch NA, et al. (2012) [8]	14	59	female	primary biliary cirrhosis	374 mg/dL	an oval-shaped change in the white matter
Rosario M, et al. (2013) [9]	15	49	female	alcoholic cirrhosis	723 μg/dL	bilateral canges in the insular and cingulate cortices, and thalami
	16	49	male	alcoholic cirrhosis	396 μg/dL	bilateral canges in the insular and cingulate cortices, and thalami
	17	40	female	drug-induced liver injury (acetaminophen)	369 μg/dL	bilateral canges in the insular and cingulate cortices, and thalami
Shinde SS, et al. (2014) [10]	18	21	male	hepatocellular carcinoma	1,032 mg/dL	not available
Mahmood T, et al. (2015) [11]	19	35	female	OTC deficiency	593 μg/dL	normal
Algahtani H, et al. (2018) [12]	20	26	female	OTC deficiency	466 μg/dL	ovale-shaped changes in the white matter, bilateral changes in the insular cortices
Reis E, et al. (2020) [13]	21	56	male	Ureaplasma infection	661 μg/dL	bilateral canges in the insular and cingulate cortices, and thalami
	22	45	male	drug-induced liver injury (acetaminophen)	>400 μg/dL	bilateral canges in the insular and cingulate cortices, and thalami
	23	49	male	cirrhosis (unknown cause)	745 μg/dL	bilateral changes in the insular and cingulate cortices
Pendela VS, et al. (2020) [2]	24	53	male	OTC deficiency	519 μg/dL	normal
our case	25	81	female	HBV-related cirrhosis	322 μg/dL	bilateral changes in the insular and cingulate cortices

The mechanism of these findings has not been fully elucidated, but a major hypothesis is that glutamine produced from ammonia causes swelling of astrocytes, resulting in brain edema. Other hypotheses include the production of the neurotoxin alpha-ketoglutaramate [14]. However, several reports have described AHE patients whose radiographic findings of the brain were within normal limits. These patients might have presented with delayed cerebral edema if they had undergone follow-up imaging tests.

Second, follow-up MR imaging is useful in detecting late-onset cerebral abnormalities. Our patient showed prolonged MR imaging abnormalities, which seemed correlated with her persistent disorientation. Treusch and colleagues described a woman with HAE who became asymptomatic two weeks after onset when her MR images also became normal [8]. Although the relationship between abnormalities on MR images and neurological prognosis has not been investigated, follow-up MR imaging may be useful in predicting neurological recovery of HAE patients. The differential diagnosis of symmetric abnormal signal intensity in MR images includes posterior reversible encephalopathy syndrome, seizure activity, and diffuse hypoxic-ischemic injury [6].

The delayed imaging findings of diseases have well been described in other fields, which can be detected by follow-up imaging tests. For example, it has been known that patients with early pneumonia may not present with significant findings on chest radiographs [15]. Follow-up chest radiography is useful in diagnosing pneumonia in some of these patients [16]. Imaging tests should, if possible, be evaluated more than once to assess the state of diseases over time.

## Conclusions

AHE can present as delayed cerebral abnormalities, and follow-up MR imaging is useful for the diagnosis of this condition. These abnormalities can be revealed in MR images several days after the serum ammonia level reaches its peak, and can be missed without follow-up imaging tests. Further reports should be accumulated to determine whether “hidden” AHE may be much more frequently present and whether follow-up imaging tests may contribute to picking up AHE patients with poor clinical outcomes.
